# Prognostic Impact of the Findings on Thin-Section Computed Tomography in stage I lung adenocarcinoma with visceral pleural invasion

**DOI:** 10.1038/s41598-018-22853-1

**Published:** 2018-03-16

**Authors:** Mei Yuan, Jin-Yuan Liu, Teng Zhang, Yu-Dong Zhang, Hai Li, Tong-Fu Yu

**Affiliations:** 1 Department of Radiology, The First Affiliated Hospital of Nanjing Medical University, Nanjing, 210009 China; 2 Department of Thoracic Surgery, The First Affiliated Hospital of Nanjing Medical University, Nanjing, 210009 China; 3 Department of Pathology, The First Affiliated Hospital of Nanjing Medical University, Nanjing, 210009 China

## Abstract

Visceral pleural invasion (VPI) in stageI lung adenocarcinoma is an independent negative prognostic factor. However, no studies proved any morphologic pattern could be referred to as a prognostic factor. Thus, we aim to investigate the potential prognostic impact of VPI by extracting high-dimensional radiomics features on thin-section computed tomography (CT). A total of 327 surgically resected pathological-N0M0 lung adenocarcinoma 3 cm or less in size were evaluated. Radiomics signature was generated by calculating the contribution weight of each feature and validated using repeated leaving-one-out ten-fold cross-validation approach. The accuracy of proposed radiomics signature for predicting VPI achieved 90.5% with ROC analysis (AUC, 0.938, sensitivity, 90.6%, specificity, 93.2%, PPV: 91.2, NPV: 92.8). The cut-off value allowed separation of patients in the validation data into high-risk and low-risk groups with an odds ratio 12.01. Radiomics signature showed a concordance index of 0.895 and AIC value of 88.9% with regression analysis. Among these radiomics features, percentile 10%, wavEnLL_S_2, S_0_1_SumAverage represented as independent factors for determining VPI. Results suggested that radiomics signature on CT exhibited as an independent prognostic factor in discriminating VPI in lung adenocarcinoma and could potentially help to discriminate the prognosis difference in stage I lung adenocarcinoma.

## Introduction

Non-small cell lung cancer (NSCLC) is the leading cause of cancer mortality worldwide^1^. Surgical resection is the treatment of choice for early stage NSCLC. Visceral pleural invasion (VPI) in NSCLC is known as a poor prognostic factor with worse survival and an important stage descriptor^2,3^. Patients with tumor invasion beyond the elastic layer (PL1), and those across the pleura with exposure on the visceral pleural surface (PL2) are combined to define VPI. In the eighth edition of TNM classification, T1 tumors were upstaged to T2 category in the presence of VPI because of its significant different prognosis^4^.

Multiple studies^5–7^ evaluated 2D and 3D CT image features in the assessment of VPI in NSCLC. Ebara *et al*.^7^ differentiated parietal from visceral pleural invasion in peripheral lung cancer that abutted to the pleura by using the ratio of tumor-pleura interfacial area to tumor size on three-dimensional CT images with accuracy of 77%. Hsu *et al*.^5^ evaluated three types of pleural tags that did not abut to the pleura, and showed that the presence of type 2 pleural tags (one or more linear pleural tag with soft tissue component at the pleural end) can help to make early diagnosis of VPI with accuracy of 71%. However, in clinical, the relations of tumor to adjacent pleura are more complicated than pleural tags and abutting. Moreover, the classifications of adjacent pleural morphologic patterns are partly inconsistent among different studies^5,6^.

Although most studies evaluated the morphologic changes of VPI on CT images, till now, no studies proved any morphologic pattern could be referred to as a prognostic factor. Besides the unsatisfactory diagnostic accuracy in previous studies, few studies investigated why in the similar distance to pleura, or even much more distant to pleura, some peripheral NSCLC have VPI, while others not. Is it corresponding to the potential malignant characteristics of primary tumors, such as the predominant subtypes, pathological grades or some potential malignant features on CT? It remains unclear whether certain prognostic impact could be used to discriminate VPI from the early stage NSCLC with 3 cm or less in size. Thus, our study was designed to integrate comprehensive information to evaluated potential malignant characteristics and prognostic factor for discriminating VPI in early-stage NSCLC.

Recent advances in radiomics enable the noninvasive evaluation of tumor internal heterogeneity by extracting and analyzing a large amounts of advanced quantitative imaging features from medical images. These high-dimensional extracted features, termed as radiomics features, could obtain a comprehensive characterization and detect potential malignant features of tumors with complex components^8^. The selection and integration of radiomics database could return a result with information about the phenotype of tumor, or clinical outcome, etc, and presents as a predicting biomarker, which was termed as radiomics signature^9–11^. Here, we hypothesize that a large number of extracted radiomics features coupled with appropriate statistical analysis may be able to detect the potential malignant characteristics of NSCLC with VPI.

As for NSCLC, squamous cell carcinoma and adenocarcinoma showed significantly different biological behaviors and prognosis^12,13^. Moreover, tumors greater than 3 cm with or without VPI showed significant different prognosis with tumor 3 cm or less in size. In order to avoid confusion and complexity, our study only evaluated the prognostic impact of lung adenocarcinoma which accounts for the most common subtypes of peripheral lung cancer and tumor size within 3 cm. Therefore, the purpose of the study was to identify the ability of multi-feature-based radiomics combined with pathological findings in differentiating the phenotypes of stage I lung adenocarcinoma with visceral pleural invasion.

## Results

### Clinical and Histopathological Findings

The final cohort was comprised of 327 patients (129 men, mean age, 62.8 years ± 13.44; 198 women, mean age, 60.3 years ± 10.83). Among them, 192 patients were diagnosed as stage IA without elastic layer invasion VPI (−) and 135 patients were stage IB with VPI (+) (81 PL1 and 54 PL2). With regard to the relation of predominant subtypes to VPI, patients with VPI (−) occurred most often in lepidic predominant adenocarcinoma (LPA) (78 of 100 [78%]), while VPI (+) occurred frequently in micropapillary (MP) (17 of 22 [77.3%]) and solid predominant subtypes (9 of 9 [100%]). The details of pathological characteristics are shown in Table 1.

**Table 1:** Summary of pathologic characteristics of included lesions.

**Variable**	**Total**	**T1a-cN0M0 Stage IA**	**T2N0M0 Stage IB**	**Χ** ^**2**^
**Sex**				
Male	129	73	56	
Female	198	119	79	
**Smoking status**				
Yes	154	84	70	
No	173	108	65	
**Tumor size**	1.51 cm(0.5–3.0)	1.35 cm (0.5–2.8)	1.72 cm (0.8–3.0)	
**Pathological grade for NSCLC**				
LPA	100(30.6%)	78(78%)	22(22%)	P = 0.000^↑^
Acinar	150(45.9%)	86(53.3%)	64(42.6%)	
Papollary	27(8.26%)	15(55.6%)	12(44.4%)	
MP	22(6.73%)	5(22.7%)	17(77.3%)	P = 0.009^↑^
Solid	9(2.75%)	0(0%)	9(100%)	P = 0.000^↑^
Mucinous	19(5.81%)	8(42.1%)	11(57.9%)	
**Pathological grade**				
Grade I	43(13.1%)	43(100%)	0(0%)	P = 0.000^↑^
Grade II	259(79.2%)	144(55.6%)	115(44.4%)	
Grade III	25(7.65%)	5(20%)	20(80%)	P = 0.003^↑^

Note. -Unless otherwise specified, data are number of lesions. *TNM stage is based on the eighth edition of the Union for International Cancer Control and American Joint Committee on Cancer TNM classification for lung cancer. LPA = lepidic predominant adenocarcinoma; MP = micropapillary. ^↑^Χ^2^ analysis showed significant difference with P < 0.05.

With regard to pathological grades, most patients were grade II (259 of 327 [79.2%]) with no difference between PL0 and PL1, PL2. For grade I (0 of 43 [0%]), patients were less frequent with VPI (+), while patients with grade III (20 of 25 [79.2%]) were more frequent with VPI (+).

There were no significant differences for age, sex, smoking status between stage IA and stage IB with VPI (+).

### Construction of the Radiomics Signature

**Figure 1:**
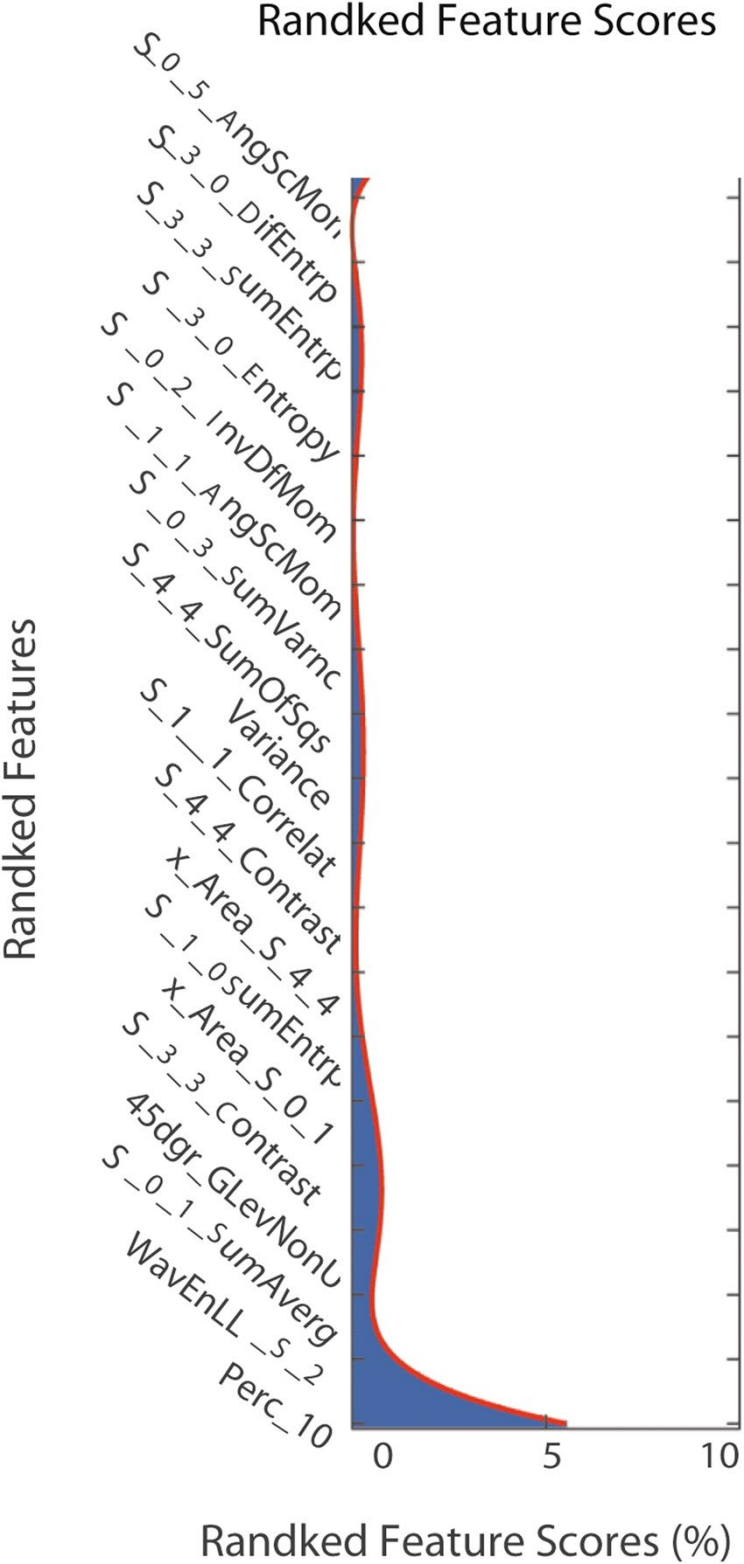
The contributions of top 19 radiomics features in the radiomics signature according to PCA analysis.

**Figure 2:**
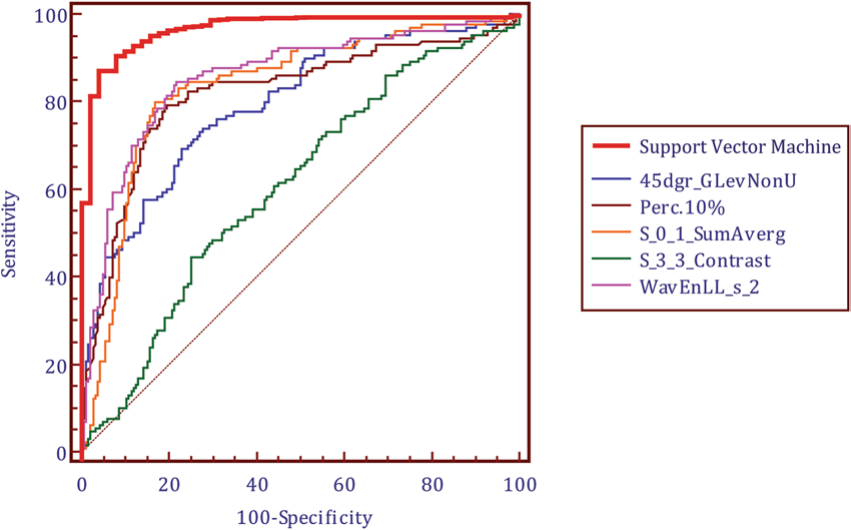
ROC analysis of the diagnostic ability of radiomics signature for distinguishing stage IA lung adenocarcinoma from stage IB with visceral pleural invasion. It showed that multiple radiomics features based signature had significantly higher accuracy than single best-performing features, all P < 0.05.

Figure 1 illustrated the top 19 best attributing radiomics features, with total weights 93.3% when accumulating these top 19 ranked features. Among them, five highest ranked radiomics features were Percentile 10%, WavEnLL_S_2, S_0_1_SumAverage, 45dgr_GLevNonU, S_3_3_Contrast, with total weights to 89.0%. The diagnostic performances of top-five best features by ROC curve analysis were illustrated in Table 2; Fig. 2. Univariate logistical analysis revealed that top four-best performing features showed significant difference between VPI (+) and VPI (−) (Table 3).

**Table 2:** Effectiveness of SVM-based Radiomics Signature in Discriminating Stage IA and Stage IB NSCLC.

	Stage IA (n = 192)	Stage IB (n = 135)	P value	Cutoff value	Auccary %	Az	SEN (%)	SPE (%)
**Radiomics signature**	0.791	1.217	0.000^↑^	>1.003	90.5	0.938^‡^	90.6	93.2
Percentile 10%	−420 ± 136	−211 ± 104	0.000^↑^	>−311	80.1	0.818	73.5	81.0
WavEnLL_S_2	1378 ± 621	3075 ± 522	0.000^↑^	>2061.1	79.8	0.856	79.5	80.8
S_0_1_SumAverage	19.20 ± 10.11	30.65 ± 6.07	0.000^↑^	>24.74	81.2	0.832	78.2	83.7
45dgr_GLevNonU	20.73 ± 15.11	98.7 ± 40.3	0.001^↑^	>49.70	72.4	0.794	70.9	78.7
S_3_3_Contrast	14.41 ± 6.36	10.01 ± 5.78	0.007^↑^	≤7.35	65.1	0.606^☨^	51.7	70.1

Note. -Unless otherwise indicated, data are mean ± standard deviation. Az = area under the receiver operating curve; SEN = sensitivity; SPE = specificity. ^↑^P < 0.05 between stage IA and stage IB NSCLC with Mann–Whitney U test. ^‡^Multiple radiomics features-based signature showed significantly higher Az value when compared with top-five performed radiomics features. ^☨^S_3_3_Contrast showed significantly lower Az value than other top-four radiomics features.

**Table 3:** Prognostic Models for Predicting NSCLC with Visceral Pleural Invasion.

**Analysis model**	**Odds Ratio**	**P value**	**Model-fitting information**	
			**AIC (%)**	**Concordance Index**
**Univariate analysis of Prodominant Subtype**	0.623	0.640	56.7	0.55
**Univariate analysis of single radiomics-based factor**				
Percentile 10%	3.914	0.000^**†**^	—	—
WavEnLL_S_2	3.181	0.002^**†**^	—	—
S_0_1_SumAverage	−2.145	0.033^**†**^	—	—
45dgr_GLevNonU	3.042	0.003^**†**^	—	—
S_3_3_Contrast	1.622	0.106	—	—
**Multivariate analysis of radiomics features**				
**Radiomics signature**	12.01	0.000	88.9	0.895^↑^
Percentile 10%	7.513	0.000^‡^	78.3	0.745
WavEnLL_S_2	2.714	0.007^‡^	70.1	0.641
45dgr_GLevNonU	4.22	0.000^‡^	68.7	0.670

Note.-AIC = Akaike information criterion. ^**†**^Potential significances were identified in four-best performing radiomics features with univariate analysis, ^‡^Percentile 10%, WavEnLL_S_2, 45dgr_GLevNonU showed to be independent factors with multiple regression analysis. ^↑^Multiple radiomics features-based signature showed significantly higher Concordance Index than single radiomics features.

### Validation of the Radiomics Signature

**Figure 3:**
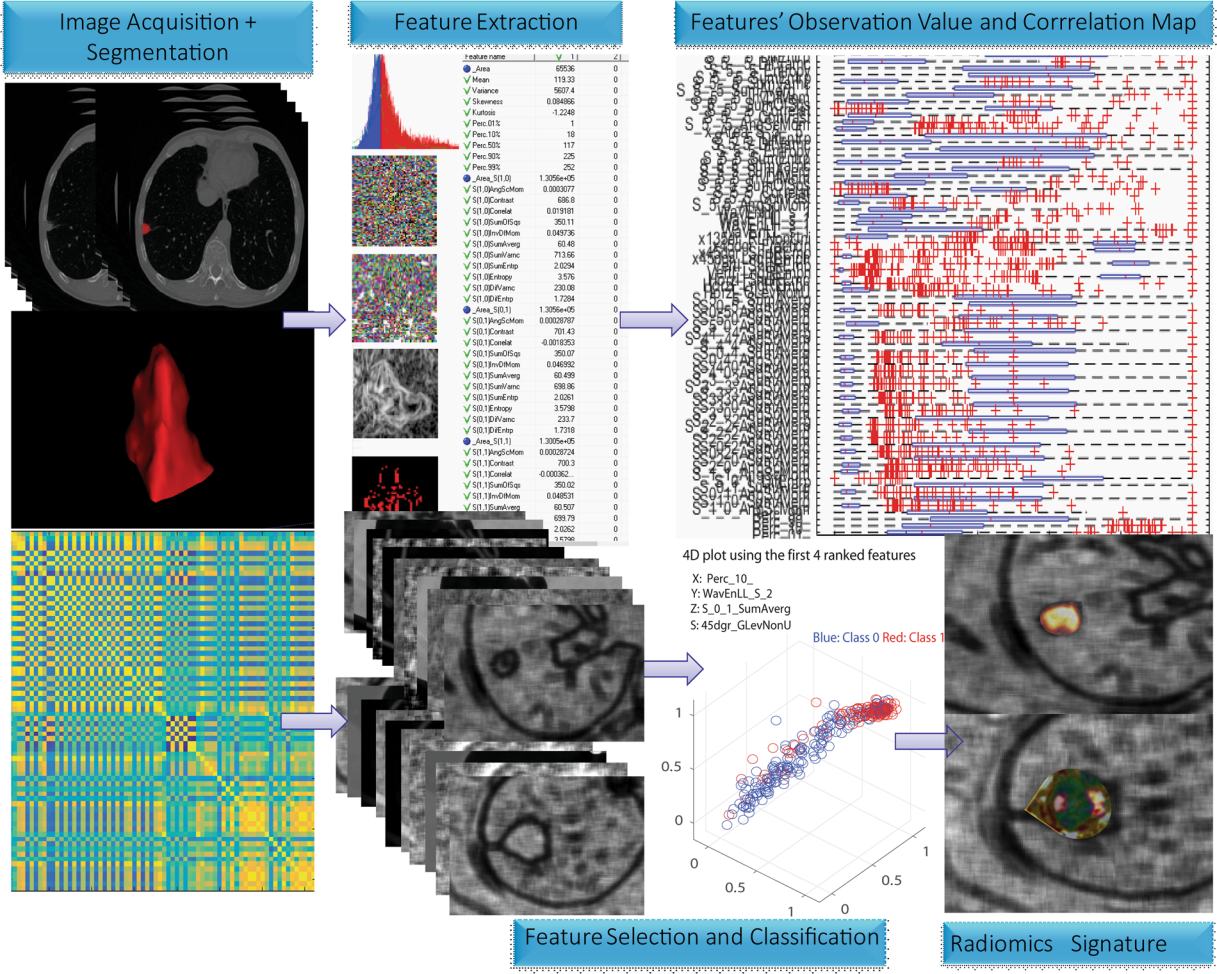
Workflow of radiomics signature generation. Radiomics features were extracted from segmented VOI on CT scanner, quantifying tumor shape, intensity, texture and wavelet features. After prioritize the features on the basis of reproducibility, redundancy, feature selection and classification, radiomics signature were generated by integrating multiple radiomics features. The cut-off value was generated by ROC analysis after modeling by SVM.

The workflow for radiomics signature generating was illustrated in Fig. 3. When using SVM for confirming the diagnostic performance in the validation cohort, the accuracy for predicting VPI (+) from VPI (−) achieved 90.5% with ROC curve analysis (AUC, 0.938, sensitivity, 90.6%, specificity, 93.2%, PPV: 91.2, NPV: 92.8, +LR: 13.4, −LR: 0.101). The optimum cut-off value generated by ROC analysis after modeling by SVMs was 1.003 with Pi value of 0.787. Accordingly, patients in the validation data were classified into high-risk and low-risk groups. The regression analysis modeled by SVM showed that the radiomics signature showed significant difference between VPI+ and VPI− groups with P = 0.000 and odds ratio 12.01. AIC value achieved 88.9% and concordance index was 0.895 in the validation cohort.

When validating each of five-best performing features, percentile 10%, wavEnLL_S_2, S_0_1_SumAverage showed to be independent factors in stratifying patients into high-risk and low-risk groups in the validation data set with wilcoxon signed-rank test. However, when compared with multiple radiomics features based signature, each of the prognostic radiomics feature showed significantly lower Az value and concordance index (Tables 2 and 3; Fig. 2).

## Discussion

Our study showed that visceral pleural invasion occurred significantly more often in MP and solid predominant subtypes and less frequently in lepidic predominant adenocarcinoma. For acinar subtype, it did not show predominance. Most of the patients included in the present study were grade II (259 of 327 [79.2%]) and showed no predominance between stage IA and stage IB. Multi-feature-based radiomics signature was identified to be an independent factor for estimating peripheral lung adenocarcinoma with VPI (+) with accuracy of 90.5%. In addition, with the cut-off value generated by ROC analysis, patients were successfully stratified into high-risk and low-risk groups, which enabled us to evaluate the risk stratifications in a quantitative and non-invasive way.

The patients with visceral pleural invasion were proved to be an independent adverse prognostic factor with potency to invade lymphangion, blood vessels and develop into metastatic disease (lymph node or distant metastasis). Evaluating the morphologic manifestation of lung adenocarcinoma with VPI on CT images is insufficient, because till now, no studies demonstrated any morphologic pattern could be referred to as a prognostic factor. Apart from the CT morphologic changes, few studies integrated comprehensive information to evaluated potential malignant characteristics and prognostic factor for the patients with VPI. To the best of our knowledge, we are the first researcher to report on the investigation of prognostic factor for predicting VPI in the early-stage lung adenocarcinoma. By analysis of the predominant subtypes of 327 stage I adenocarcinoma, we found that patients with VPI occurred significantly more often in MP and solid subtypes, which proved to be with poor prognosis^14,15^. However, the sample size for MP and solid subtypes are limited in stage I lung adenocarcinoma. With regard to the pathological grade, most of the patients were grade II with no predominance between stage IA and IB. The predictive value of the predominant subtypes and pathologic grade to discrimination between VPI (+) and VPI (−) appeared to be limited.

The extraction of advanced radiomics signature allowed us to quantitatively assess the heterogeneous internal features of lung adenocarcinoma with different tumor phenotypes on a macroscopic tissue scale by converting imaging data into high dimensional quantitative descriptors^13^. Intratumor heterogeneity calculated by radiomics has been suggested to correlate with worse clinical outcome, greater risk for lymph node involvement and distant metastasis^8,13,16^, and should be able to demonstrate the potential malignant characteristics of primary lung adenocarcinoma with VPI. Consistent with the hypothesis, the present study showed that integrated radiomics cut-off value of the patients in stage IB lung adenocarcinoma with VPI (+) was significantly higher than the patients in stage IA VPI (−), and the radiomics signature was validated as a prognostic factor in the validation data. These findings supported that radiomics signature may infer the tumor phenotypic characteristics in stage I lung adenocarcinoma and discriminate the potential malignant characteristics of primary adenocarcinoma with VPI.

The radiomics signature included five-best performing radiomics features: percentile 10%, wavEnLL_S_2, S_0_1_SumAverage, 45dgr_GLevNonU, S_3_3_Contrast, which represented intratumor heterogeneity within different radiomics feature groups. All features showed significant difference between stage IA and IB with VPI, which are partly consistent with results of recent studies on prognostic stratification^16^. Among them, percentile 10%, wavEnLL_S_2, S_0_1_SumAverage were identified as independent factor in the validation data set with wilcoxon signed-rank test. Among them, percentile 10% represents the point at which, 10% of the voxel values that form the histogram are found from the left. WavEnLL_S_2, which is a label for wavelet feature, is the energy of wavelet coefficients in subband LL. S_0_1_SumAverage is one of the labels for the co-occurrence matrix features: values in parenthesis represent coordinates, containing information about distance and direction between pixels. All these features represented intratumor heterogeneity within different radiomics groups. However, when compared the diagnostic performance of single radiomics-based factor with multiple radiomics features-based signature, radiomics signature showed significantly higher in Az value and concordance index, indicating that high dimensional radiomics factors should be integrated to select valuable biomarkers for phenotypic characteristics, which is similar to genomics.

Our study has several limitations. Firstly, the sample size is relatively small and lack of an external validation. Secondly, although radiomics demonstrated the intratumor heterogeneity within invasive adenocarcinoma, whether these macroscopic imaging features have underlying biologic relevance is not clear and should be investigated further. Thirdly, disease free survival in the study is based on the previous studies. We did not evaluate the difference of 5-year disease-free survival rate between stage IA and stage IB with VPI (+) in lung adenocarcinoma, because the median follow-up time is short considering that treatment failures may occur up to several years. The survival rate is insufficient for statistics. Further validation should be done according to the survival.

In conclusion, a multi-feature-based radiomics signature by thin-section CT was designed to identify tumor-phenotypes of lung adenocarcinoma with visceral pleural invasion. The new radiomics biomarker exhibited as an independent prognostic factor in discriminating VPI and may provide a non-invasive opportunity for evaluating the prognosis in early-stage lung adenocarcinoma.

## Materials and Methods

### Patients

This study was approved by the institution of the First Affiliated Hospital of Nanjing Medical University (Nanjing, China) and all methods were carried out in accordance with the approved guidelines. All subjects provided written informed consent to participate in the study. We systematically reviewed 739 patients with peripheral lung adenocarcinoma who underwent chest CT scans with thin-section (1.0 mm) images from the period of January 2014 to December 2016. Inclusion criteria were as follows: (a) all the patients underwent surgical resection and diagnosed by pathologic examination; (b) pathological-N0M0 peripheral lung adenocarcinoma 3.0 cm or less in greatest dimension according to the eighth edition of TNM staging system^4^; (c) thin-section CT scan was performed within 90 days before surgery; (d) available results for clinical data, including age, sex, smoking history, *et al*. A total of 327 Asiatic patients met all the inclusion criteria and 412 patients were excluded because of one or more of the following: (a) CT scan with intravenous administration of contrast materials (n = 180); (b) unsatisfactory imaging quality due to respiratory artifact during examination (n = 41); (c) pathological invasion to parietal pleura (PL3) (n = 50); (d) tumor size >3 cm (n = 100); (e) associated with separate tumor nodule as the primary tumor or directly invades any of the following structures: chest wall, phrenic nerve, parietal pericardium, diaphragm, mediastinum, heart, great vessels, trachea, recurrent laryngeal nerve, esophagus, vertebral body, and carina (n = 41).

### CT scanning

All the patients underwent unenhanced chest CT with 64-silce (Definition) or 128-slice (Definition AS+; Siemens, Malvern, Pa) row CT scanner with 1.0 mm slice-thickness and 0.8 mm reconstruction interval. The protocol was as follows: 100–120 kVp, mAs were set based on CARE Dose4D for exposure dose reduction. All images were reconstructed with a high-kernel (b60) with 512 × 512 matrix. Window settings: standard lung (window width, 1500 HU; window level, −600 HU) and mediastinum (window width, 350 HU; window level, 50 HU).

A pathologist with 10 years experiences who was blinded to the imaging findings evaluated the histopathologic patterns and T, N descriptors according to eighth edition TNM staging system^4^. Elastic stains were performed to clarify the status of VPI when initial hematoxylin and eosin stained slides showed that the tumors were adjacent to the pleura. VPI was classified according to the eighth edition of TNM classification: PL0 (T1) as lack of pleural invasion beyond the elastic layer, PL1 (T2) as invasion beyond the elastic layer, PL2 (T2) as invasion to the surface of the visceral pleura and PL3 (T3) as invasion of the parietal pleura. The differences in survival were statistically significant between PL0 and PL1, PL2 for either ≤3 cm or >3 cm in size. However, there were no statistically significant differences in survival between PL1 and PL2^3^. Thus, in the present study, PL1 and PL2 were combined into VPI (+) group and were upstaged to T2a in those lesions ≤3 cm in size, and PL0 were classified into VPI (−) group. We evaluated the difference of histopathological and CT radiomics features between these two groups.

### Segmentation and morphological features extraction

**Figure 4:**
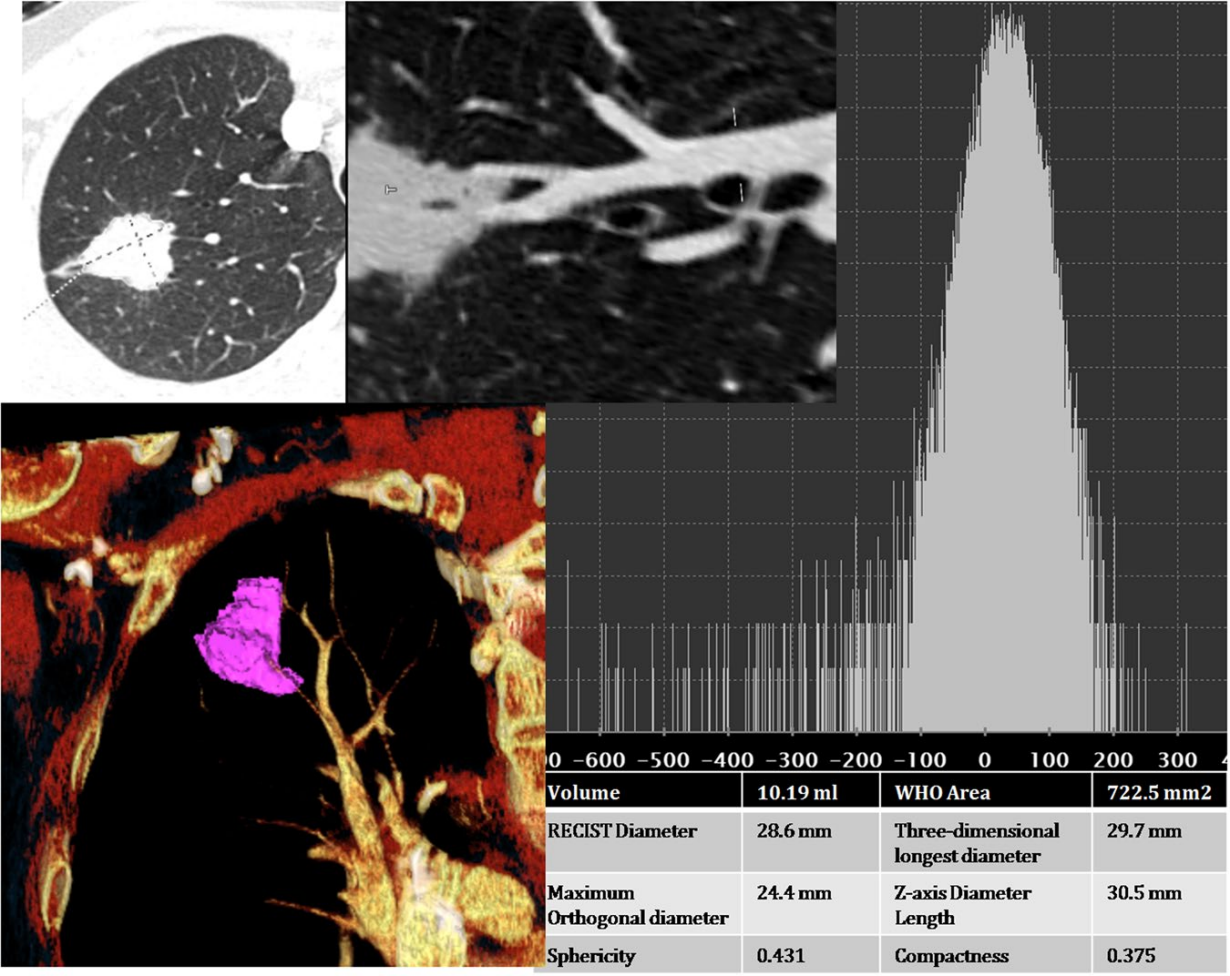
Example computed tomography (CT) images in a patient with stage IB lung adenocarcinoma. Axial longest diameter of the lesion was placed manually and the contour of entire-tumor volume of interest (VOI) was automatic segmented by LungCAD. Morphological features were extracted from the defined tumor contour on CT images.

Each nodule was automatic segmented by running on lungCAD software (Siemens SOMATOM Force CT). Firstly, two thoracic radiologists (author 2 and author 3 with 8-years and 6-years of experience in chest imaging, respectively) who were blinded to the pathologic result independently placed the longest diameter of the lesion. Secondly, precise edge of the entire-tumor volume of interest (VOI) was autosegmented by lungCAD. Visually identified mismatching, blood vessels and the chest wall adjacent to the margin of nodule were manually adjustment. Three-dimensional longest diameter and other 7 morphological features were computed separately by lungCAD (Fig. 4).

### Radiomics features extraction and selection

A total of 308 radiomics features were extracted and quantified by using AnalysisKit (GE Healthcare, China) for tumor phenotypes from whole VOI segmented previously. Two experienced radiologists who performed lesion segmentation independently extracted these features. Radiomics features are divided into four groups: I) shape, II) tumor intensity, III) texture, IV) wavelet features. Tumor intensity was estimated by using histogram analysis with 9 features. Then, 271 texture features, derived from the gray level co-occurrence (GLCM) and run length matrices (GLRLM), were extracted from CT scans. Finally, the coiflet wavelet transformation was used to compute 20 wavelet features, which are the transformed domain representations of the intensity and textural features.

Feature selections on the basis of reproducibility and redundancy were performed to prioritize these high-dimensional features. Firstly, concordance correlation coefficient (CCC) was used to test the reproducibility and stability of each imaging feature. Top 100 most stable features with CCC value ≥0.9 were kept. The equation is described as:

1 $${{\rm{\rho }}}_{{\rm{c}}}=\frac{2{{\rm{\rho }}{\rm{\sigma }}}_{{\rm{x}}}{{\rm{\sigma }}}_{{\rm{y}}}}{{{\rm{\sigma }}}_{{\rm{x}}}^{2}+{{\rm{\sigma }}}_{{\rm{y}}}^{2}+{({{\rm{\mu }}}_{{\rm{x}}}-{{\rm{\mu }}}_{{\rm{y}}})}^{2}}$$

where μ_x_ and μ_y_ are mean values of variance x and y; σ_x_, σ_y_ are mean squares; andρrepresents the correlation coefficient of x and y.

Then we removed redundant features with nearest neighbor distance <0.05. Eighty-three features were kept after adjusting redundancy.

### Feature Selection and Classification

In the present study, we implemented a robust recursive feature elimination (RFE) method based on SVM for feature selection. The RFE-SVM was performed to create an integrated radiomics data and returned a result with ranking features by recursively training on SVM. An iterative method was performed and the feature with smallest ranking score (contribution weight ω) was removed until cumulative ω of all desired features reached 80%.

SVM with radical basis function (RBF) kernel was applied for separating the labeled training data into two classes. The SVM classifier is considered as a supervised learning task, which projects the data into multidimensional space to separate two classes with a hyperplane. For SVM with RBF kernel, the equation is described as:

2 $${\rm{k}}({\rm{x}},{{\rm{x}}}_{{\rm{i}}})=\exp (-{\rm{\gamma }}{|{\rm{x}}-{{\rm{x}}}_{{\rm{i}}}|}^{2})$$

where x and x_i_ are two input vectors, and Gamma (γ) controls the shape of the hyperplane.

As patient numbers were relatively small, SVM classifiers were trained (cohort 1) and validated (cohort 2) using repeated (10 repeat iterations) and leaving one out ten-fold cross validation approach, in which, except one fold for validation(cohort 2), the other nine folds were applied for training (cohort 1). This procedure was repeated until each case in the database was used once in the validating set.

As the direct output value of classifiers does not show probabilities of VPI, we converted the output values to the probabilities (Pi) by applying a sigmoid function as follows:

3 $$Pi=\frac{1}{1+{e}^{-{\rm{x}}}}$$

where *x* is the output value of classifiers. The value of Pi, which indicates the probabilities that the target lesion has VPI, was also termed as radiomic signature, as it integrated a multi-feature based radiomics information and indicated a cut-off point for the probability of VPI phenotype.

### Performance Evaluation and Statistical Analysis

We validate the predictive performance in the validation cohort using Receiver Operating Characteristic (ROC) regression curve and quantified by using the areas under the ROC curves (AUC), referring to the method of DeLong *et al*.^17^. Diagnostic accuracy, sensitivity, specificity, positive predictive value (PPV), negative predictive value (NPV), positive likelihood ratio (+LR) and positive likelihood ratio (−LR) were calculated. Regression model of radiomics signature was generated and Akaike information criterion (AIC) was used as a measure of goodness of fit. Concordance index was used to assess the prognostic capability of radiomics signature (concordance index, 0–1).

Univariate and multivariate logistic regression analysis was used to determine the prognostic factor of radiomics and significant radiomics features. Wilcoxon signed-rank test was used to validate the performance of single best-performing feature.

All statistical analyses were performed with statistical packages (SPSS 17.0 Chicago, III; MedCalc software, version 8.2.0.1, Mariakerke, Belgium). Standard PCA was completed by SPSS software (PASW Statistics 22.0; SPSS Inc., Chicago, IL, USA). P < 0.05 was considered to indicate a statistically significant difference.

## References

[1] Siegel RL, Miller KD, Jemal A (2017). Cancer Statistics, 2017. CA Cancer J Clin.

[2] Yoshida J (2009). Visceral pleura invasion impact on non-small cell lung cancer patient survival: its implications for the forthcoming TNM staging based on a large-scale nation-wide database. J Thorac Oncol.

[3] Kawase A (2010). Visceral pleural invasion classification in non-small cell lung cancer. J Thorac Oncol.

[4] Rami-Porta R, Asamura H, Travis WD, Rusch VW (2017). Lung cancer - major changes in the American Joint Committee on Cancereighth edition cancer staging manual. CA Cancer J Clin.

[5] Hsu JS (2016). Pleural Tags on CT Scans to Predict Visceral Pleural Invasion of Non-Small Cell Lung Cancer That Does Not Abut the Pleura. Radiology.

[6] Qi LP (2016). Multivariate Analysis of Pleural Invasion of Peripheral Non-Small Cell Lung Cancer-Based Computed Tomography Features. J Comput Assist Tomogr.

[7] Ebara K (2015). Pleural invasion by peripheral lung cancer: prediction with three-dimensional CT. Acad Radiol.

[8] Huang Y (2016). Radiomics Signature: A Potential Biomarker for the Prediction of Disease-Free Survival in Early-Stage (I or II) Non-Small Cell Lung Cancer. Radiology.

[9] Lambin P (2012). Radiomics: extracting more information from medical images using advanced feature analysis. Eur J Cancer.

[10] Kumar V (2012). Radiomics: the process and the challenges. Magn Reson Imaging.

[11] Hawkins S (2016). Predicting Malignant Nodules from Screening CT Scans. J Thorac Oncol.

[12] Schuurbiers OC (2014). Glucose metabolism in NSCLC is histology-specific and diverges the prognostic potential of 18FDG-PET for adenocarcinoma and squamous cell carcinoma. J Thorac Oncol.

[13] Wu J (2016). Early-Stage Non-Small Cell Lung Cancer: Quantitative Imaging Characteristics of (18)F Fluorodeoxyglucose PET/CT Allow Prediction of Distant Metastasis. Radiology.

[14] Lee HY (2012). Solitary pulmonary nodular lung adenocarcinoma: correlation of histopathologic scoring and patient survival with imaging biomarkers. Radiology.

[15] Sica G (2010). A grading system of lung adenocarcinomas based on histologic pattern is predictive of disease recurrence in stage I tumors. Am J Surg Pathol.

[16] Aerts HJ (2014). Decoding tumour phenotype by noninvasive imaging using a quantitative radiomics approach. Nat Commun.

[17] DeLong ER, DeLong DM, Clarke-Pearson DL (1988). Comparing the areas under two or more correlated receiver operating characteristic curves: a nonparametric approach. Biometrics.

